# Dietary Modification Combined with Nutrition Education and Counseling for Metabolic Comorbidities in Multiple Sclerosis: Implications for Clinical Practice and Research

**DOI:** 10.1007/s13668-024-00538-8

**Published:** 2024-04-27

**Authors:** Shoroog Allogmanny, Yasmine Probst

**Affiliations:** 1 School of Medical, Indigenous and Health Sciences, Faculty of Science, Medicine and Health, University of Wollongong, Wollongong, NSW 2522 Australia; 2 Clinical Nutrition Department, College of Applied Medical Sciences, Taibah University, Madinah, 42353 Saudi Arabia

**Keywords:** Multiple sclerosis, Metabolic comorbidities, Nutrition, Education and counseling, Behavior

## Abstract

**Purpose of Review:**

Metabolic comorbidities such as obesity, diabetes, hypertension, and dyslipidemia are common to multiple sclerosis (MS) and are associated with negative outcomes of the disease. Dietary intervention has the potential to improve MS co-morbidities; thus, it is a high priority for people living with MS to self-manage their disease. The present review aimed to summarize the recent evidence on the impacts of combining dietary modification with nutrition education and counseling on managing metabolic comorbidity markers in MS.

**Recent Findings:**

Evidence suggests important roles for tailored dietary change strategies and nutrition education and counseling in managing metabolic comorbidities for MS. There is also indirect evidence suggesting a relationship between dietary fiber, the gut microbiome, and improved metabolic markers in MS, highlighting the need for more research in this area. For people living with MS, addressing both barriers and facilitators to dietary changes through behavior change techniques can help them achieve sustainable and tailored dietary behavior changes. This will support person-centered care, ultimately improving metabolic comorbidity outcomes.

**Summary:**

Metabolic comorbidities in MS are considered modifiable diseases that can be prevented and managed by changes in dietary behavior. However, the impact of targeted dietary interventions on mitigating MS-related metabolic comorbidities remains inadequately explored. Therefore, this review has provided insights into recommendations to inform future best practices in MS. Further well-designed studies based on tailored dietary strategies applying behavior change theories are needed to address the underlying determinants of dietary practice in this population.

## Introduction

Multiple sclerosis (MS) is an increasingly prevalent inflammatory demyelinating disease of the central nervous system (CNS) [1]. MS results in a heterogeneous array of unpredictable debilitating symptoms, including sensory disturbances (e.g., tingling, numbness, and itching), physical challenges (e.g., fatigue, spasticity, muscle weakness, and loss of balance), cognitive impairment (e.g., memory loss and poor concentration), emotional symptoms (e.g., depression and anxiety), and vision problems (e.g., blurred vision and pain in eye movement) [1, 2]. However, the manifestation and severity of the symptoms vary among individuals depending on the location of demyelination in the CNS [3].

Besides the presence of symptoms, comorbidities are more common in people with MS than in the general population [4•, 5]. Comorbidities in MS refer to the presence of additional medical conditions that coexist alongside MS and require treatment [6]. A growing body of literature highlights metabolic comorbidities such as obesity, diabetes, hypertension, and dyslipidemia are common in MS and are risk factors for cardiovascular disease (CVD) [4•, 7–9]. CVD, hypertension, dyslipidemia, and diabetes are grouped as “vascular comorbidities” [10]. Increasing evidence has demonstrated  that vascular comorbidities in MS are linked to greater diagnostic delays [11], higher severity of common symptoms [12], an increasing rate of healthcare utilization [13], economic burden [14], and higher mortality rates [15].

A recent retrospective cohort study showed that hypertension, CVD, and type 2 diabetes (T2DM) are associated with an accelerated progression of disability in MS, as measured by the Expanded Disability Status Scale (EDSS) [8]. This is because metabolic comorbidities, particularly T2DM, are connected to reduced brain and grey matter volume [15]. Obesity, which is highly prevalent in people with MS, is related to an increased risk of additional comorbidities [16]; thus, it has been associated with worsening MS symptoms and disease progression [7, 17]. Another study has found that people with MS who have dyslipidemia and three or more comorbidities have a higher relapse incidence over 2 years compared to those without comorbidities [18]. Consequently, metabolic comorbidities result in a lower health-related quality of life (HRQoL) in people with MS [4•, 19].

Sustainable and tailored strategies are needed to delay the onset and manage metabolic comorbidities and their effects on MS outcomes. Studies have shown that metabolic comorbidities are often considered modifiable diseases, which can be preventable or modified by adopting healthy lifestyle behaviors [20–22]. Healthy lifestyle practices include improved eating habits, regular physical activity, maintaining a healthy weight, limited alcohol consumption, and smoking cessation. Incorporating healthy lifestyle behaviors to optimize MS outcomes such as metabolic comorbidities, is of increasing interest [23]. Dietary modification, as one element of healthy lifestyle behaviors, is a high priority for people with MS to self-manage their disease [24–26].

While there is emerging evidence of the role of dietary modification as a non-pharmacological treatment in MS, MS-specific nutrition guidance is lacking [27–29]. Dietary recommendations for people with MS are based on general population guidelines that recommend following a balanced diet to optimize overall health and decrease the risk of diet-related comorbidities [27, 28]. In the general population, changes in dietary habits are considered a first-line strategy for modulating and delaying the onset of metabolic comorbidities. For example, changes toward a healthy diet among people at risk of metabolic comorbidities have been linked with reduced risk of T2DM, CVD, and microvascular complications [30], as well as improved glucose homeostasis and blood lipid profiles [31]. Likewise, evidence from observational studies highlighted that adherence to a higher-quality dietary pattern with mild-to-moderate alcohol intake is associated with a lowered risk of metabolic comorbidities and improved HRQoL in people with MS [21, 32•] because of the potential of protecting the neurologic reserve [33]. By contrast, adherence to a less healthy  diet is linked with a higher risk of obesity, an altered blood lipid profile, the presence of at least two metabolic comorbidities, and a worsened EDSS in MS [34].

In addition to early prevention, it is well-Established that already-present metabolic comorbidities can be managed by dietary interventions in the non-MS population [35]. Incorporating nutrition education and counseling by utilizing behavior change techniques can also improve markers of metabolic comorbidities [36]. Behavior change techniques include active components employed in an intervention to support and facilitate desirable changes in dietary habits [37]. However, the role of dietetic interventions in MS-related metabolic comorbidities are yet to be reviewed. Therefore, this review was conducted to summarize the available literature on dietary modification and nutrition education and counseling and their impact on metabolic comorbidity markers in MS. Metabolic markers include the body mass index (BMI), body weight (BW), waist circumference (WC), blood pressure, blood lipid profile, fasting blood glucose, hemoglobin A1c (HbA1c), and insulin.

## Literature on Dietary Modification, Nutrition Education and Counseling, and Metabolic Comorbidities in MS

### Dietary Intervention-Related Metabolic Comorbidity Markers in MS

Rimmer et al. [38] conducted a 9-month trial that involved a tailored, telehealth weight management program. The program included a personalized healthy diet delivered via videos, telephone consultations, and educational resources. The intervention was effective in reducing BW and BMI (*p* = 0.04) among 27 adults with physical disabilities (including MS). Another trial of nutrition counseling for people with MS (*n* = 57) incorporated education on healthy eating habits and regular messages on a WhatsApp group [39••]. The participants also received a collection of educational booklets, dietary records, and measuring tools for cooking to help them cook and choose appropriate foods. After 3 months of monthly counseling sessions, the intervention group showed significant improvements in the anthropometric measurements, including BW, WC, and BMI, as well as dietary intake assessed by a 24-h recall (all *p* < 0.05). Thus, the intervention led to significant weight loss among participants who were considered to be overweight or obese (*p* < 0.05).

These findings are consistent with another 3-month feasibility study that focused on behavioral dietary changes based on a low glycemic load (GL) diet [40••].  The diet focused on healthy dietary patterns (i.e., whole foods with low GI and minimally processed foods). The study applied a digital health approach, including education delivered via weekly modules, weekly tele-coaching calls, and the use of a mobile app to record dietary intake daily. After the intervention, participants with MS (*n* = 18) exhibited an improvement in cardiometabolic risk, including HbA1c, fasting blood glucose, blood pressure, and body composition. Another study by Papandreou et al. [41••] evaluated a 3-month dietitian education and counseling program that included individualized dietary interventions based on the Mediterranean diet (MedDiet) for 20 women living with MS. A significant (*p* < 0.001) decrease in BW, BMI, fat mass, and serum glucose was observed compared to the baseline, which was negatively correlated with cholesterol intake levels (*p* < 0.05). The MedDiet pattern is based on a high intake of fruits, vegetables, whole grains, olive oil, seeds, and legumes, a moderate intake of dairy products, and a low intake of animal fats [42].

Yadav et al. [43] conducted a 1-year-RCT that allocated participants into either a diet with dietitian counseling (*n* = 26) or a control group (*n* = 27). The findings revealed that a diet based on complex starchy carbohydrates without animal products or vegetable oils significantly (*p* < 0.001) reduced BMI. In addition, several serum metabolic biomarkers significantly decreased in the intervention group compared to the control; these biomarkers included total cholesterol (*p* = 0.027), low-density lipoprotein (LDL) cholesterol (*p* = 0.031), and fasting insulin levels (*p* = 0.0068). However, an inconsistent result was found in another intervention [44], where 40 mins of nutrition education once per  month for 3 months, teaching 34 people with MS about a healthy diet and practical strategies to purchase, prepare, and cook food did not result in changes to BMI or WC, although the quality of the diet did improve, as assessed by the Healthy Eating Index (HEI)-2010.

 To date, existing interventions have mainly focused on tailored healthy lifestyle changes with an emphasis on higher diet quality to improve markers of metabolic comorbidities in MS. Nutrition education and counseling are part of a tailored approach that is associated with increased adherence to dietary guidelines [45]. However, data on dietary adherence are limited in the reviewed interventions. Thus, exploring long-term outcomes such as the adherence to and sustainability of changes to dietary behavior are  essential to inform future best practices in MS care.

### Gut Microbiome, Metabolic Comorbidities, and Implications for MS

The reviewed intervention studies emphasized limiting the intake of highly processed foods and increasing the consumption of fruits, vegetables, legumes, grains, and other sources of dietary fiber. Three studies reported favorable outcomes on metabolic markers and found that fiber intake significantly increased after the intervention [38, 39••, 41••]. This is consistent with the dietary guidelines that recommend a well-balanced, diverse, and fiber-rich diet. Nutrition is a modifiable element that is able to shape the composition and characteristics of the gut microbiome [46]. A fiber-rich diet leads to gut eubiosis (a balance in the gut microbiota) through increased gut bacteria diversity and anti-inflammatory mediators associated with microbes [46, 47]. This results in reducing the risks of obesity, hyperlipidemia, hyperglycemia, T2DM, and CVD in the general population [48]. On the other hand, a low-fiber, Western diet increases the levels of pro-inflammatory mediators and promotes gut dysbiosis (an imbalance in the gut microbiota) [46, 49], which disturbs the metabolic markers [50]. Emerging evidence indicates that alterations in the composition and function of the gut microbiome may lead to the development and progression of metabolic comorbidities [50].

Compared to healthy individuals, people with MS experience gut dysbiosis more often, which may promote systemic inflammation [51]. Thus, emerging evidence leans  toward the role of the gut microbiome in modulating CNS inflammation and its implications for MS pathogenesis [51, 52]. This may provide indirect evidence of the influence of dysbiosis on the pathogenesis of metabolic comorbidities in MS, though research focused on the intricate interplay between the diet, gut microbiome, and metabolic comorbidities in MS is limited. As a result, future MS dietary trials may benefit from collecting microbiota samples to elucidate the role of dietary modification on  the gut microbiome and metabolic comorbidity outcomes in MS. This may lead to novel therapeutic avenues for slowing MS progression and optimizing clinical outcomes by addressing metabolic comorbidities.

### Nutrition Education and Counseling: Individualized Approach for Best Practices

**Fig. 1:**
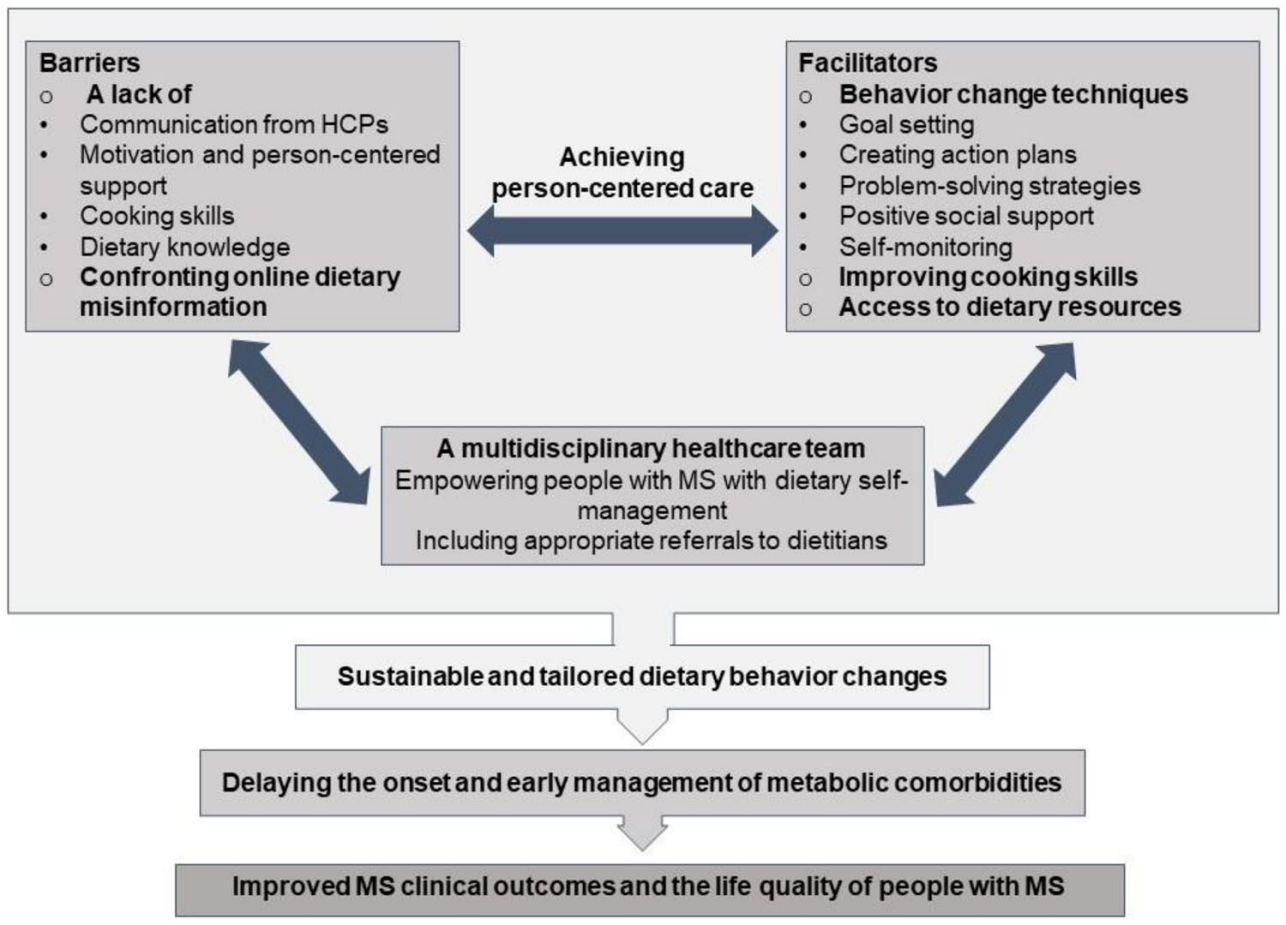
Producing sustainable and tailored dietary behavior changes for improving metabolic comorbidities in MS by achieving person-centered care

The following section discusses improving metabolic comorbidities in MS by achieving person-centered care through addressing both barriers and facilitators to dietary changes, depicted in Fig. 1.

Modifiable comorbidities in MS can be recognized in the context of chronic disease care models [10], which involve the responsibility of healthcare professionals (HCPs) to provide education in routine care. This aims to promote self-management, ultimately preventing or  managing comorbidities. Nutrition education and counseling were incorporated into the reviewed interventions to empower and motivate participants to attain dietary self-management through behavioral changes. An individualized approach is required because of the complexity of MS [53]. Changes in the dietary behavior of people with MS are also complex and need ongoing support [54]. Accordingly, using appropriate behavior change theories to guide interventions is recommended to guide  best practice for ensuring sustainable changes in dietary behavior [55]. Nonetheless, only one intervention reported the use of a behavior change framework, which was the Health Action Process Approach (HAPA) behavior change theory to guide implementation of the activities [40••].

The inclusion of behavior change techniques that promote personalization goals, self-regulatory skills, and social engagement while anticipating personal barriers may improve adherence to dietary changes [56]. A large body of qualitative evidence has explored barriers to achieving self-management behavioral changes, including a healthy diet in people with MS. Barriers identified were related to a lack of motivation, person-centered support, and communication from HCPs [57, 58]. Additional barriers to adopting healthy dietary choices include a lack of cooking skills and dietary knowledge [59] as well as online dietary misinformation [26]. Behavior change techniques were integrated into the reviewed interventions to overcome these barriers and promote positive changes in dietary behavior. One intervention was informed by motivational interviewing techniques to support patient-centered care [38]. Other techniques utilized were goal setting and creating action plans [38, 40••], problem-solving strategies [40••], social environment support [39••, 44], addressing food preparation and cooking [38, 39••, 44], and providing practical dietary resources [38, 39••, 40••, 44]. Self-monitoring to encourage individuals to regularly track their dietary changes were  also employed in the interventions [38, 39••, 40••].

Integrating evidence-based nutrition education into the routine care of people with MS, who may face unique physical and cognitive l challenges due to the disease, is crucial. The interventions reviewed were delivered and facilitated by dietitians [40••, 41••, 43] and coaches with health education and kinesiology degrees [40••]. Ideally, nutrition educators are dietitians; however, nutrition counseling can be limited by low referral rates, the cost of dietetic services, or remote locations [60]. Hence, changes in behaviors, including nutrition, must become basic competencies for all HCPs who provide support for people living  with/or at risk of nutrition-related chronic diseases [61]. Neurologists and a multidisciplinary team in MS care are needed to empower people with MS with dietary behavior self-management near the time of diagnosis and across all stages of the disease, including appropriate referrals to dietitians care. For interventions to be equitable and sustainable, dietary guidance should be personalized and tailored to the individual’s needs, barriers should be identified, and  strategies to facilitate changes should be provided to this population. This will ensure tailored changes in dietary behavior and will result in person-centered care, delaying onset or  early management of metabolic comorbidities, and in turn, improving clinical outcomes and quality of life for people with MS.

## Conclusion and Recommendations for Research

Metabolic comorbidities such as obesity, diabetes, hypertension, and dyslipidemia are considered modifiable lifestyle diseases that can be prevented early and managed in MS by dietary modification as an adjunct to first-line therapies. Prevention and intervention of metabolic comorbidities through dietary management in people with MS should target behavioral changes for sustainable outcomes. Furthermore, dietetic management should be incorporated as part of a team-based approach in routine MS care to improve the outcome of the disease.

While current evidence suggests that dietary interventions play a role in improving metabolic comorbidity markers in MS, the impact of targeted dietary interventions on mitigating metabolic comorbidities remains inadequately explored. The current evidence is constrained by the paucity of intervention studies, small sample sizes, and the short duration of the interventions. To address this gap, further well-designed trials that include tailored dietary behavior modification strategies are needed. Interventions based on behavior change theories are also required to address the underlying determinants of dietary practices in people with MS. Together, these can improve our understanding of the role of tailored dietary behavior modification in managing and decreasing the prevalence of metabolic comorbidities in MS.

## Data Availability

Not applicable.
